# New insight into protein glycosylation in the development of Alzheimer’s disease

**DOI:** 10.1038/s41420-023-01617-5

**Published:** 2023-08-25

**Authors:** Jingwei Zhao, Minglin Lang

**Affiliations:** 1 CAS Center for Excellence in Biotic Interactions, College of Life Science, University of Chinese Academy of Sciences, Beijing, 100049 China; 2 College of Life Science, Agricultural University of Hebei, Baoding, 071000 China

**Keywords:** Alzheimer's disease, Alzheimer's disease

## Abstract

Alzheimer’s disease (AD) is a chronic neurodegenerative disease that seriously endangers the physical and mental health of patients, however, there are still no effective drugs or methods to cure this disease up to now. Protein glycosylation is the most common modifications of the translated proteins in eukaryotic cells. Recently many researches disclosed that aberrant glycosylation happens in some important AD-related proteins, such as APP, Tau, Reelin and CRMP-2, etc, suggesting a close link between abnormal protein glycosylation and AD. Because of its complexity and diversity, glycosylation is thus considered a completely new entry point for understanding the precise cause of AD. This review comprehensively summarized the currently discovered changes in protein glycosylation patterns in AD, and especially introduced the latest progress on the mechanism of protein glycosylation affecting the progression of AD and the potential application of protein glycosylation in AD detection and treatment, thereby providing a wide range of opportunities for uncovering the pathogenesis of AD and promoting the translation of glycosylation research into future clinical applications.

## Facts

- The glycosylation patterns of some key AD proteins are altered in AD brains, such as APP, tau and transferrin, etc.
- Altered protein glycosylation may contribute to the production and deterioration of AD through multiple pathways.
- New modalities for diagnosis and treatment of AD targeting protein glycosylation are being developed.
- N-glycosylation, O-glycosylation and O-GlcNAc glycosylation have different effects on protein structure and function.

## Open Questions

- Is altered protein glycosylation a cause or a consequence of AD?
- Given the complex inter-regulatory relationship among N-glycosylation, O-glycosylation and O-GlcNAc glycosylation, what is the key factors that precisely regulated their changes throughout the progression of AD?
- How to design drugs that target specific protein glycosylation sites for treating AD?

## Introduction

Alzheimer’s disease (AD) is a chronic neurodegenerative disease and the most common cause of dementia in old adults with symptoms including a serious decline in memory and short-term learning ability, language dysfunction, and short lifespan, etc. [1]. Currently, more than 50 million people worldwide are affected by dementia, of which about 70% are caused by AD [2]. With the increase of the ageing population, the incidence of AD is increasing year by year. The disease seriously endangers the physical and mental health and the quality of life of the elderly, not only causing severe pain to patients themselves but also bringing a heavy burden to their families and society. How to diagnose and cure AD effectively has become an urgent scientific question and a serious social problem, which attracted widespread attention from the medical profession and governments around the world.

AD can be categorized into early-onset AD (EOAD, onset before age 65) and late-onset AD (LOAD, onset after 65) according to the age of onset [3]. EOAD accounts for about 5%-6% of all AD cases, and patients with EOAD will face a greater risk for death compared to those with LOAD (when controlling for the direct effect of aging on death). There are differences in clinical symptoms between early-onset AD and late-onset AD. Patients with EOAD are more likely to have language, visuospatial, or dysexecutive phenotypes rather than the amnestic disorder commonly seen in LOAD [4]. It is worth noting that EOAD is not equivalent to familial AD. However, there may be an association between EOAD and familial AD based on statistical data [5].

The neuropathological features of AD include the formation of extracellular Aβ neurotic plaques and intracellular neurofibrillary tangles (NFTs) [1]. Aβ is produced through the sequential cleavages of its precursor protein APP by β-secretase and γ-secretase and then it aggregates to form amyloid plaques or diffusible soluble oligomers, which are thought to be more toxic [5]. Aβ plays an important role in neurotoxicity and neural function. In addition to cognitive impairment, the accumulation of dense plaques in the hippocampus, amygdala, and cortex leads to synaptic damage and loss [6]. Tau is a microtubule-associated protein that promotes microtubule assembly and maintains microtubule stability. NFT refers to the abnormal filaments of hyperphosphorylated tau proteins that intertwine with each other to form paired helical filaments (PHF) and accumulate in the perinuclear cytoplasm, axons, and dendrites, resulting in impaired physiological function, apoptosis and neuronal loss [7]. In addition, brain atrophy caused by loss of nerves, nerve cells, and synapses can also provide evidence for AD progression [1].

Studying the pathogenesis of AD is of great significance for its clinical diagnosis and treatment. Up to now, there are two mainstream hypothesizes for AD pathogenesis, the cholinergic hypothesis and the amyloid hypothesis [1]. Other hypotheses include tau misfolding propagation hypothesis [8], neuroinflammation hypothesis [9], metal ion disorder hypothesis [10], etc. Table 1 listed the main contents of each hypothesis [11–13]. However, no widely accepted theory to explain the pathogenesis of AD at present.

**Table 1:** Hypothesis of AD pathogenesis.

Hypothesis	Main content	Reference
Cholinergic hypothesis	The decline in acetylcholine (Ach) concentration and cholinergic activity leads to damage to cholinergic neurons	[11]
Amyloid hypothesis	APP is wrongly cleavaged to produce Aβ40 and Aβ42, the formation of Aβ amyloid fibrils induce neurotoxicity and tau pathology	[12, 13]
Tau misfolding propagation hypothesis	The aggregated extracellular Tau enters cells and transmits a misfolded message specifically to intracellular Tau and induce its fibrillization	[8]
Neuroinflammation hypothesis	Immune-mediated autodestructive processes may occur in AD, triggering cellular inflammatory response and lead to cell death	[9]
Metal ion disorder hypothesis	Disturbance of metal ion metabolism in AD may cause cognitive deficits and affect oxidative stress leading to neuronal death	[10]

In recent years, an increasing number of studies have uncovered a new clue that the glycosylation patterns of some key AD-associated proteins were altered in the patients’ brains, suggesting an interesting link between glycosylation and the pathogenesis of AD [14–16], and spurring extensive interest of scientists and entrepreneurs. Further researches on the relationship between protein glycosylation and AD may bring new insight into understanding the causes of AD. Moreover, the abnormally glycosylated proteins in AD can be used as biomarkers for clinical diagnosis of AD and potential targets for AD treatment, which provide an opportunity for promoting the translation of glycosylation research into clinical applications.

Briefly, glycosylation is one of the most common post-translational modification of proteins by which a carbohydrate is covalently attached to a target protein and affect various functions of the protein. The role of carbohydrates as structural components is important in the construction of complex multicellular organs and organisms [17]. The common monosaccharides involved in the formation of sugar chains on human glycoproteins include glucose (Glc), galactose (Gal), mannose (Man), N-acetylgalactosamine (GalNAc), fucose (Fuc), N-acetylglucosamine (GlcNAc), N-acetylneuraminic acid (Neu5Ac). The types of sugar chains on glycoproteins are classified according to the properties of the chemical bonds between them and the proteins to which they are attached, mainly including N-glycans and O-glycans. N-glycans are generally classified into types of high mannose, complex and hybrid. The sugar chain of N-glycans has a common pentasaccharide core composed of GlcNAc and Man, which is usually attached to the Asn residues in Asn-X-Ser/Thr consensus sequence in the polypeptide chain where X ≠ Pro. O-glycans mainly refer to the glycans with sugar chain linked to the Ser/Thr residues of the peptide chain through GalNAc, which contain a variety of different type of core structures. There are also sugar chains of O-glycans linked through mannoses. O-glycosylation occurs in the Golgi apparatus [17, 18], while O-GlcNAc glycosylation is a special kind of O-glycosylation modification which distinguished it from the usual protein O-glycosylation. Protein O-GlcNAc glycosylation refers to the process by which individual GlcNAc are added to serines and threonines. This process usually takes place in the cytoplasm and is synthesized by the involvement of O-linked GlcNAc transferase (OGT) and O-linked GlcNAc glycosylase (OGA). Interestingly protein O-GlcNAcylation has been found to play a non-negligible role in AD development [15].

**Fig. 1:**
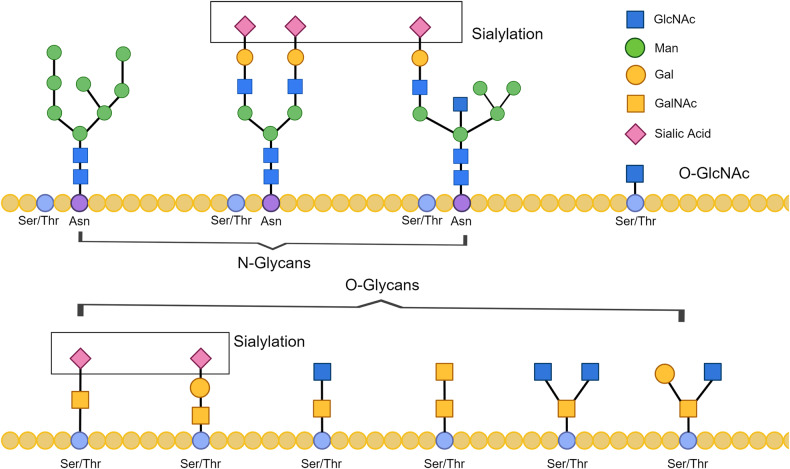
Common glycan structures in AD. The sugar chain of N-glycans is linked to the Asn-X-Ser/Thr consensus sequence in the polypeptide chain and has a common pentasaccharide core composed of GlcNAc and Man. The sugar chain of O-glycan is linked to Ser/Thr in the polypeptide chain and has no fixed core structure. The O-GlcNAc is a single GlcNAc being added to the Ser/Thr in the polypeptide chain. Sialylation modifies the glycan chains in the N-/O-glycan.

Sialic acid is usually present at the terminal position of the protein glycan chain. Sialylation, the modification of adding sialic acid to the sugar chain, is mediated by sialyltransferases (ST) and takes place in the Golgi apparatus [15]. Since protein sialylation alterations seem to be continuously emphasized in AD-related studies, the uniqueness of sialylation as a glycan chain modification modality is deliberately highlighted here. Figure 1 shows a schematic diagram of the glycan structures corresponding to the forms of protein glycosylation mentioned above (N-/O-/O-GlcNAcylation/sialylation).

Several reviews have been dedicated to summarize the progress of studies related to the abnormal protein glycosylation in AD, providing an overview of this field in terms of the impact of glycosylation at different loci on proteins and the potential of glycosylated proteins in the diagnosis and treatment of AD [14, 15, 19]. This review focuses on the effects of various AD key proteins that modified by their different glycosylation patterns, i.e., N-glycosylation/O-glycosylation/O-GlcNAcylation/sialylation, and tries to generalize the overall roles played by the different altered glycosylation patterns in the development of AD pathology. We also highlighted the diagnostic tools and therapeutic agents for AD that target specific protein glycosylation and that have been experimentally proven to be feasible. Our review will hopefully engage researchers beyond the glycosylation field to study this fascinating topic, encouraging breakthroughs in this area.

### Impaired protein glycosylation is linked to ad progression

In the cells of AD patients, the glycosylation pattern of various proteins was found to be altered, including the location and quantity of glycosylation, suggesting which may play critical roles in the progression of AD. Multiple genes related to glycosylation have also been found to be aberrantly expressed in AD cells, including a group of glycosylation genes that may augment or ameliorate tauopathy phenotypes [20]. Increased levels of GnT-III mRNA expressing enzymes that synthesize bisected GlcNAc residues were found in AD brain cells, consistent with another study finding that N-glycans carrying bisected GlcNAc structures were increased in AD brains [21, 22]. However, recent evidence suggests that the content of N-glycans carrying bisected GlcNAc structures in AD brain varies by brain region, and the content of this glycan is decreased in the hippocampus [23]. A proteomic study in the cerebrospinal fluid (CSF) of AD patients finds a decreasing trend in fucosylation and an increasing trend of endogenous peptide O-glycosylation in CSF [24]. Altered glycosylation patterns are detected for a number of N-glycoproteins including α−1-antichymotrypsin, ephrin-A3, and carnosinase CN1 [25]. Fucosylated and bisecting GlcNAc structures are more abundant in the brain cells of females with AD [26]. Recent studies have found alterations in the glycosylation of low-abundance proteins such as C1-inhibitor and α−1-acid glycoprotein in AD brain cells [27]. Significant changes in the levels of protein O-GlcNAcylation and N-/O-glycosylation are detected in the brain, CSF, and serum of AD patients. But it is worth noting that the same changes are not applicable in brain cells of patients with other neurodegenerative diseases [28], suggesting that new sugar-based biomarkers may become potential specific pathogenic indicators of AD and be applied in AD diagnosis.

In order to better understand AD and thus diagnose and cure it, the pathogenesis of AD has been continuously investigated in recent years [19, 29–31]. There is currently no accepted answer to the mechanism by which glycosylation affects AD pathogenesis. However, it has been experimentally demonstrated that protein N-glycosylation is involved in multiple dysregulated processes and pathways in AD brain, including extracellular matrix dysfunction, neuroinflammation, synaptic dysfunction, cell adhesion alteration, lysosomal dysfunction, endocytic trafficking dysregulation, endoplasmic reticulum dysfunction, and cell signaling dysregulation [32]. O-glycosylation has also been shown to be closely related to neuronal plasticity in AD, including axonal and dendritic sprouting and reactive synaptogenesis [31]. These findings provide strong support for further studies on the mechanism of glycosylation affecting AD pathogenesis. Based on the effects of glycosylation on protein function, activity, lifespan, etc., there have been many specific speculations about the role of glycosylation in the pathogenesis of AD. Studying the mechanism of glycosylation affecting the pathogenesis of AD can deepen our understanding of AD, and may be beneficial to the design of drugs and courses of treatment for AD by changing the glycosylation patterns.

There have been various speculations and clinical evidence for the mechanism of the generation of two major pathological features of AD: the deposition of Aβ amyloid plaques and forming neurofibrillary tau tangles. And the role of abnormal protein glycosylation in the development of AD has received further attention in recent years, which is becoming a new feature of AD. The important proteins in AD pathology, such as APP and tau, have significantly different glycosylation patterns from those in normal cells, and the altered glycosylation patterns occur during multiple stages of protein maturation [29, 33–35] in which glycosylation is thought to alter the processing, distribution, and trafficking of these proteins. Abnormal protein glycosylation also modulates the receptors function of signaling pathways such as Wnt by affecting their localization and stability on lipid membranes, leading to abnormal signal transduction and inducing the development and progression of AD [36]. The glycosylation pattern of some other proteins such as TREM2 and reelin that play a role in cell signaling is also altered [30, 37]. It has also been shown that protein glycosylation can alter immune processes, induce inflammatory responses in the brain, and affect the development of AD [38]. Glycomics studies, on the other hand, have quantitatively demonstrated changes in the frequency of overall protein glycosylation in AD brains as well as specific alterations in the glycosylation patterns of a large number of proteins and mapped glycoprotein profiles in different brain regions [32, 39, 40]. The development of glycoproteomic technologies has provided global-level evidence for altered glycosylation in AD.

### Impaired glycosylation of signature proteins app and Tau

#### App

**Fig. 2:**
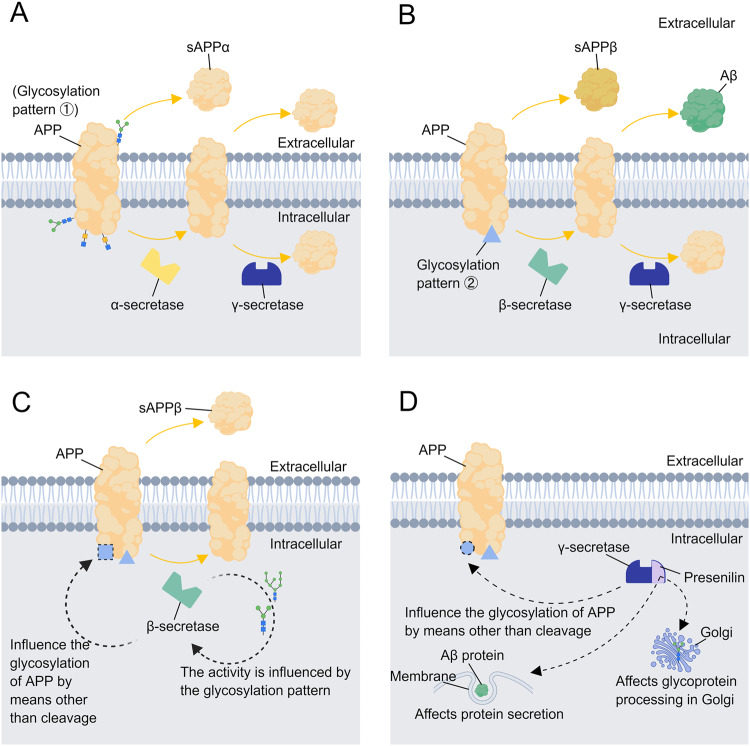
Glycosylation affects the formation of Aβ protein. **A**, **B** APP can be cleaved successively by α-secretase and γ-secretase or β-secretase and γ-secretase. The latter leads to the secretion of Aβ protein, which aggregates to form plaques or more toxic oligomers. APP that undergoes two different cleavage patterns differs in glycosylation. **C** Except for cleaving APP, β-secretase also affect the sialylation and complex N-glycosylation of APP. And the activity of β-secretase can be influenced by its glycosylation pattern. **D** Except for cleaving APP, γ-secretase also affect the processing of its complex N-glycosylation in Gorgi as well. Its component, presenilin, affects the ability of glycoprotein production of Golgi and the secretion of several proteins including Aβ protein. Each of the blue shapes (triangles, circles, squares) on the APP in the Fig. 2C and 2D represent a different glycosylation pattern.

APP is first cleaved by α-secretase in healthy human cells to produce an extracellular domain secretory fragment (sAPPa) and a membrane-bound carboxy-terminal fragment. γ-secretase then cleaves the carboxy-terminal fragment into small fragments that can be fully degraded, i.e., the non-amyloid pathway. In the development of AD, APP is cleaved successively by β-secretase and γ-secretase to generate Aβ fragments of different lengths. Aβ aggregates to form plaques or more toxic oligomers. This processing is called the amyloid pathway [41]. The mRNA expression of APP was significantly upregulated in AD, and the sAPPα glycosylation pattern was also altered compared to normal cells. The study by Tomita *et al*. found that most of the cleavage of APP by α, β, and γ secretase occurs after APP is modified by O-glycosylation in the Golgi apparatus [42], which implies the pathway through which APP is processed and hydrolyzed may be influenced by its glycosylation pattern, resulting in different products. Further studies confirmed this claim. The lectin binding assay determined that APP cleaved by α-secretase and β-secretase have different lectin binding patterns, i.e., APP that undergoes two different cleavage patterns differs in glycosylation [43] (Fig. 2A and 2B). The result proved that glycosylation determines the processing and hydrolysis pathway of APP protein. Notably, Tyr10 glycosylated Aβ peptides are significantly increased in the cerebrospinal fluid of AD patients, suggesting that sialylated O-glycans may affect APP processing [33]. Another study confirmed that O-glycosylation on this phenolic hydroxyl group on Tyr681 also affects APP processing and suggested that this effect may arise from the induction of APP conformation by such O-glycosylation [44]. Due to the central role of Aβ proteins in AD pathogenesis, the effect of glycosylation on APP hydrolysis and processing is considered a reasonable speculation about how abnormal glycosylation leads to AD development. Moreover, glycosylation may also affect Aβ protein secretion in AD. In several experiments in which targeted mutagenesis was used to delete two potential N-linked glycosylation sites of APP, N467 and N496, significantly slower initial APP secretion. A large number of APPs lacking carboxy-terminal fragments were present in the cell and the level of secreted Aβ was elevated. This experiment suggests that abnormal N-glycosylation may affect the processing and transport of APP. The N-glycosylation pattern may also affect the intracellular sorting of APP from Golgi to lysosomes [45, 46].

As for why APP is abnormally glycosylated in AD cells, studies have shown that in addition to the sequentially cleaving of APP, β-secretase and γ-secretase can also affect the sialylation and complex N-glycosylation of APP through mechanisms other than cleavage [15]. Since the glycosylation pattern of the APP protein itself also affects the types of secretases that process and hydrolyze it, implying that there is a complex inter-regulation relationship between APP and a variety of secretases. Interestingly, the activity of β-secretase can also be influenced by its glycosylation pattern (Fig. 2C). Charlwood et al. changed the four N-glycosylation sites of the Asp-2 protein (a transmembrane aspartic protease expressed in the brain, which is shown to have beta-secretase activity) and expressed it in cells. The protease activity of the mutants was then assessed using a peptide substrate and the protease activity of all three double-site mutants had been significantly reduced. This confirms that the protease activity of Asp-2 depends on its glycosylation site occupancy [47]. In addition, Aβ proteins appear to be able to influence the glycosylation of sAPPα and sAPPβ, suggesting a feedback-like effect of APP processing products on their precursors. Glycosylation similarly affects the function of γ-secretase. Inhibition of N-glycosylation of γ-secretase using the curcumin derivative GT863 revealed unchanged activity but inhibited Aβ production, demonstrating the feasibility of γ-secretase as a target for the treatment of AD [48]. In addition, presenilin, an important component of γ-secretase, affects the glycosylation pattern of AD-related proteins such as reelin and NCAM [15]. The overexpression of either wild-type or mutant PS1 disturbs glycoprotein processing within the Golgi [49], while the deficiency of PS leads to impaired secretion of Aβ proteins [50] (Fig. 2D).

Ideas on the inhibition of Aβ protein production by means to alter glycosylation have mainly focused on the modulation of enzymes that process APP, such as the curcumin derivative GT863 mentioned above. In addition, previous inhibitors designed for BACE1 with β-secretase activity often interfere with its processing of other substrates, producing severe side effects. Selective modulation of BACE1 cleavage activity towards APP by altering the glycosylation of BACE1 is considered as a promising AD therapeutic modality [51]. The regulation of BACE1 glycosylation can be achieved by the regulatory action of various glycosylases such as galactosyltransferases and mannosidases.

#### Tau

Tau protein is a microtubule-associated protein, whose hyperphosphorylation leads to abnormal tau accumulation and fibril entanglement, resulting in the formation of intracellular neurofibrillary tangles (NFTs), a classic neuropathological feature of AD. Researchers have found the existence of aberrant glycosylation of tau protein in AD patients at an early stage. Monosaccharide composition analysis and specific lectin blots showed that most of these glycosylations are N-glycosylation [29], which may have an impact on the subsequent abnormal phosphorylation of tau [52]. In contrast, the level of O-GlcNAc glycosylation of proteins in tau-rich cytoskeletal fractions produced by AD brain samples is significantly reduced [34]. In the neurofibrillary tangles formed by hyperphosphorylated tau, higher levels of O-glycosylation are detected than that in the normal human brain [35]. These results suggest that changes in the patterns of protein glycosylation may occur at multiple stages of tau formation.

**Fig. 3:**
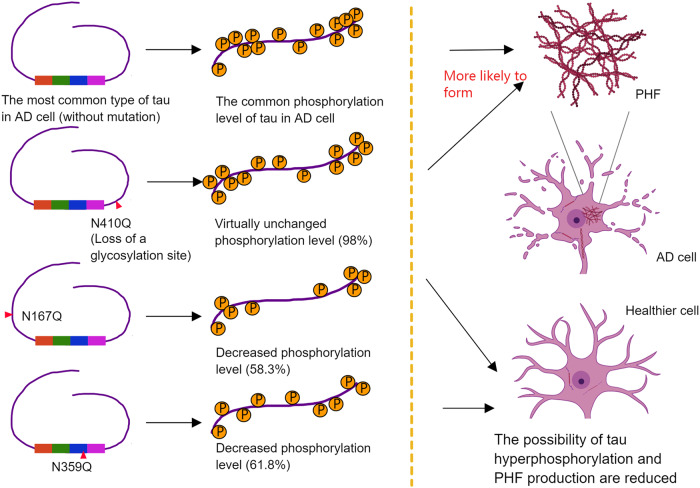
Early N-glycosylation shows a positive regulatory effect on tau phosphorylation, and may affect the further tangles of tau. When an amino acid of tau is altered from N to Q so that it cannot be glycosylated at that site, the level of tau phosphorylation is altered thereafter. The phosphorylation level of N167Q decreased to 58.3% of that of the control group and the phosphorylation level of N359Q decreased to 61.8%. However, N410Q had nearly no effect (98%). Tau with lower levels of phosphorylation are less likely to form PHF.

Aberrant glycosylation of tau protein in AD brains may also be one of the mechanisms for inducing AD pathological features. As mentioned above, the time and types of tau protein glycosylation are varied, and different glycosylation has different effects on the abnormal phosphorylation and tangles of tau. Abnormal N-glycosylation first occurs in the early stage of tau protein in AD. In vitro studies have shown that this N-glycosylation can facilitate the subsequent abnormal hyperphosphorylation of tau in AD brain [29]. Further studies have also confirmed that the phosphorylation levels of tau with N-glycosylation site mutation (N167Q, N359Q, and N410Q) are site-dependently regulated [52], revealing a positive regulatory effect of early N-glycosylation on tau phosphorylation (Fig. 3). Experiments targeting O-GlcNAc glycosylation of tau protein showed that the level of O-GlcNAc glycosylation of hyperphosphorylated tau protein was significantly reduced, suggesting an overall negative regulation of O-GlcNAc glycosylation on tau protein phosphorylation. However, it is noteworthy that the effect of O-GlcNAc glycosylation on tau protein phosphorylation exhibits significant site-specificity [53]. One speculation claims that it is because the O-GlcNAc glycosylation sites on tau overlapping with the phosphorylation site may create a competitive relationship. But for the phosphorylation site that does not overlap with the O-GlcNAc glycosylation site, the O-GlcNAc glycosylation at side sites may alter the protein conformation, making it more susceptible to phosphorylation. Then tau is further intertwined with each other to form paired helical filaments (PHFs), the main fibrous structure of NFT, in which glycosylation is thought to be responsible for the formation and maintenance of PHF structures in vivo. The study by Wang et al. showed that PHFs in AD are highly glycosylated, and the deglycosylation of PHF tangles converts them into straight filament bundles and restores their accessibility to microtubules [54]. Further studies found that PHF tangles from AD brain tissue are associated with lectin Galanthus nivalis agglutinin (GNA)-positive glycan molecules [35]. However, unlike the glycosylation patterns mentioned above, O-GlcNAc glycosylation, in turn, regulates tau aggregation and slows down tau fibrillation assembly [55]. The reason why tau protein is abnormally glycosylated in AD is unclear, but there are several hypotheses, including changes in the subcellular location of tau in AD leading to changes in its interaction with glycosylase, the increased activity of oligosaccharyltransferase (OST) in the dystrophic neurons in AD brain and the presence of a hitherto unidentified cytoplasmic N-glycosidase whose activity is down-regulated in AD brain due to certain factors [15].

The possible role of abnormally glycosylated tau proteins as targets in the detection and treatment of AD is gaining attention. As an important protein involved in the formation of PHF, the level of tau in the brain reflects the severity of AD. In a recent study, researchers designed an enzyme-linked multi-well plate assay to quantify N-glycans binding to the lectin Phaseolus vulgaris Erythroagglutinin (PHA-E). They found that the binding level of PHA-E was correlated visibly with phosphorylated tau levels and total tau levels in CSF. This finding indicates the feasibility of judging the degree of AD development by detecting the total tau level in the brain through CSF analysis [22]. Furthermore, compared to the patients with mild cognitive impairment (MCI) and AD dementia, this correlation is most pronounced in the group of patients with subjective cognitive impairment (R = 0.53–0.54), suggesting that this analysis is more suitable for patients who have not yet been diagnosed with AD to test their possibility of developing AD.

**Fig. 4:**
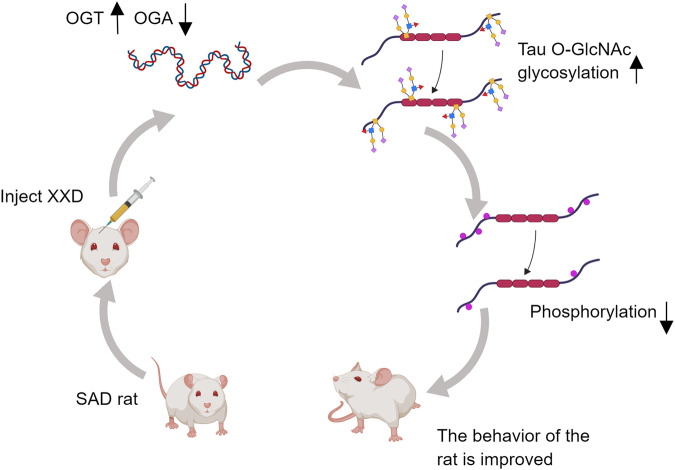
Injecting XXD improves the behavior of SAD rats. XXD significantly enhanced the expression of OGT and decreased the expression of OGA in the hippocampus of SAD rats. Therefore, the O-GlcNAc glycosylation level of tau protein in the rats treated with XXD was improved. Due to the overall inhibitory effect of O-GlcNAc glycosylation on tau phosphorylation, SAD rats treated with XXD showed reduced levels of tau phosphorylation and improved AD symptoms. This finding confirms that it is feasible to treat AD by altering protein glycosylation patterns with drugs.

A feasible idea to improve AD symptoms is to increase the level of O-GlcNAc glycosylation of tau protein, thereby inhibiting its hyperphosphorylation. O-GlcNAc glycosylation and phosphorylation of tau are generally inversely correlated. Diwu *et al*. found that the behaviors of the SAD rats treated with XXD as well as the O-GlcNAc glycosylation level of their tau proteins were dose-dependently improved and increased, respectively [56], accompanied with an enhanced expression level of OGT and a decreased expression level of OGA in the hippocampus of the treated rats [57] (Fig. 4). This finding confirms that it is feasible to treat AD by altering protein glycosylation patterns with drugs. Another substance, chicoric acid (CA), has also been shown to regulate glucose metabolism by increasing GluT1, promoting the glycosylation of tau proteins, thereby reducing their phosphorylation levels and preventing or improving AD symptoms [58]. Recently, a three-dimensional structural model of tau predicted by ab-initio modeling provided 25 potential sites for tau O-GlcNAc glycosylation, which may be a target and reference for AD drug development [59]. Some proteins whose glycosylation patterns are significantly altered in AD, such as APP, β-secretase, and tau proteins, also have considerable potential as therapeutic targets for AD for the same reason.

### Impaired glycosylation of key proteins for metal ion metabolism and oxidative stress in AD patients

Glycosylation may also affect AD pathogenesis by altering oxidative stresses. Oxidative stress is one of the important pathogenesis of AD and can participate in the development of AD by promoting Aβ deposition, tau hyperphosphorylation and the subsequent loss of synapses and neurons [60]. Transferrin (Tf) is a glycosylated metal-binding serum protein consisting of a polypeptide chain with 679 amino acid residues folded into two domains, each of which contains a metal-binding site. Transferrin transports iron to cells through the blood. Due to genetic variation, transferrin exists in various sequence forms, the most common of which is TfC1. When the proline at position 570 in TfC1 is replaced by a serine, the resulting variant is called TfC2. TfC2 has pro-oxidative properties, but the reason for this property remains unclear. The allele frequency of the TfC2 variant becomes higher in AD patients, and this change is thought to be associated with diseases that are caused by free radicals [61]. The level of glycosylation of transferrin is altered in AD. Serum analysis of two types of AD patients (TfC1 homozygote and TfC1C2 heterozygote) found that TfC1 protein sialylation levels were elevated while TfC2 protein sialylation levels were lower in AD patients [62–64]. Glycosylation can regulate the lifespan of proteins. In vivo experiments in rats have shown that the half-life of Tf proteins without sugar chains is significantly lower than that of Tf proteins containing complex glycans [65], that is, less glycosylated proteins are degraded from the cell relatively quickly. This means that the TfC2 protein in AD cells can be removed more quickly, which is beneficial to the cells. However, given the elevated pro-oxidative properties of TfC2, its comprehensive impact on AD progression remains unclear, but changes in TfC2 in AD are largely thought to promote oxidative stress. Changes in the sialylation levels of transferrin in AD suggest that glycosylation may affect protein function by regulating protein lifespan, thereby regulating oxidative stress in cells. In addition, although glycosylation of transferrin has no effect on its ability to bind iron [64], glycosylation may indirectly regulate iron homeostasis by altering iron transport by affecting the lifespan of Tf.

Two different glycosylation patterns of transferrin Tf are present in CSF and it seems that one comes from the brain while the other from the serum [66]. The ratio of these two sugar isoforms may serve as marker information for the diagnosis of AD, leading to an easy way to diagnose AD. The high-throughput lectin-TfAb ELISA assay has been shown to discriminate differences in serum Tf glycosylation levels between patients with iNPH (idiopathic normal pressure hydrocephalus, a form of senile dementia associated with ventriculomegaly) and controls [67], and has potential as a diagnostic tool for AD. The unique glycosylation pattern of Tf isomers in the CSF of AD may also serve as an indicator for the detection of AD. The Tf isomer carrying a unique mannosylated glycan (Man-Tf) is significantly elevated in the CSF of AD. Experiments have shown that combined detection of p-tau and Man-Tf levels showed approximately 90% diagnostic sensitivity and specificity for AD [68].

Other enzymes involved in oxidative stress have also been found to be regulated by glycosylation. Both in vitro and in vivo experiments confirmed that the activity of Cu, Zn-superoxide dismutases (Cu, Zn-SODs) was decreased due to their elevated glycosylation levels [69]. This enzyme is an antioxidant metalloenzyme with copper and zinc ions as prosthetic groups, which can catalyze the disproportionation of superoxide anion free radicals to generate oxygen and hydrogen peroxide, and play a key role in the balance of oxidation and antioxidant in the body [70]. The level of Cu, Zn-SOD is also affecting the intracellular metal homeostasis, which is well correlated with the metal ion disorder hypothesis for the pathogenesis of AD. Furthermore, glycosylation can modulate the activity of metalloenzymes, indicating that there may be a direct or indirect interaction between glycosylation and metal homeostasis. Post-translational modifications, including glycosylation of other antioxidant enzymes such as glutathione peroxidase and thioredoxin reductase, can also lead to loss or reduction of enzymatic activity under pathological conditions, resulting in oxidative stresses [69].

### Impaired glycosylation of proteins associated with cell signaling in AD patients

#### Reelin

The glycoprotein Reelin plays an important role in the development of the correct cell structure and organization of the central nervous system (CNS) cells. It is a key molecule regulating neuronal migration and may be involved in signal pathways related to neurodegeneration. The Reelin protein can bind to transmembrane receptors located on the adjacent cells, triggering a tyrosine kinase cascade [71]. Reelin signaling may be involved in regulating tau phosphorylation and processing of Aβ protein precursors through a series of cascade reactions and interacts with AβPP [72, 73]. Aβ may alter the glycosylation of reelin and affect its ability to bind to its receptors [74]. Recent studies have shown that the expression levels of Reelin protein are upregulated in the brain and CSF in several neurodegenerative diseases including AD, supporting the association of the Reelin protein with AD pathogenesis. In AD development, reelin signalling may be compromised due to reduced efficiency of reelin protein-receptor binding and then a vicious cycle leading to AD deterioration will form, but there is no consensus on the exact mechanism [75]. The binding degree of Reelin protein to *Lens culinaris agglutinin* (LCA) in the CSF of AD patients is significantly different from that of normal people, which proves that the glycosylation pattern of the Reelin proteins in AD is changed. Furthermore, Reelins from CSF and plasma have different binding properties to lectins such as *Lens culinaris agglutinin* and *Ricinus communis agglutinin* (RCA120), suggesting that CSF and plasma Reelins have different cellular origins [30]. The glycosylation pattern of Reelin protein affects its thermal stability and ability to interact with other proteins, and may therefore affect AD development [76].

#### AChE and BuChE

Acetylcholinesterase (AChE) is a key enzyme in the biological nerve conduction process. AChE can degrade the neurotransmitter acetylcholine between cholinergic synapses, terminate the excitatory effect, and maintain the normal transmission of nerve signals in the body [77]. AChE promotes the development and regeneration of neurons. There are various molecular forms in the human body, and the glycosylation patterns of different molecular forms of AChE are different [78, 79]. Isoelectric analysis of CSF from AD patients showed that the molecular form and distribution of AChE were significantly altered in AD [80]. Lectin binding analysis showed that the binding mode of AChE to two lectins, *concanavalin A* and *wheat germ agglutinin*, in the CSF of AD patients was significantly different from that of the control group. This difference originates from the altered ratio of AChE isomers with different glycosylation patterns in AD. Aberrantly glycosylated AChE may originate from brain cells, as similar glycosylation differences were observed in the frontal cortex of AD patients, but not in the cerebellum [81].

AChE is considered having specific AD diagnostic value due to its increased minor isoforms and altered glycosylation levels detected in the CSF of patients in AD but not in other dementias [82]. In further studies, butyrylcholinesterase (BuChE) glycosylation was also found to be altered in CSF of AD. It is thought to be over 90% sensitivity and specificity to identify AD cases by combining an analysis of CSF AChE and BuChE glycosylation [83]. Therefore, the researchers further studied whether their glycosylation isomers Glyc-AChE or Glyc-BuChE can be used as early markers of AD. Although Glyc-AChE and Glyc-BuChE levels in CSF of AD are confirmed to be positively correlated with the disease duration, and similar to tau, phosphorylated tau, and numerous Aβ proteins, and their levels are significantly higher than those in controls, however, it was found that their levels are not significantly elevated in the early stages of AD [84]. Therefore, the glycosylated isomers of AChE and BuChE are not suitable as early markers of AD, but their values as markers in judging AD disease progression cannot be ignored.

#### TREM2-R47H

**Fig. 5:**
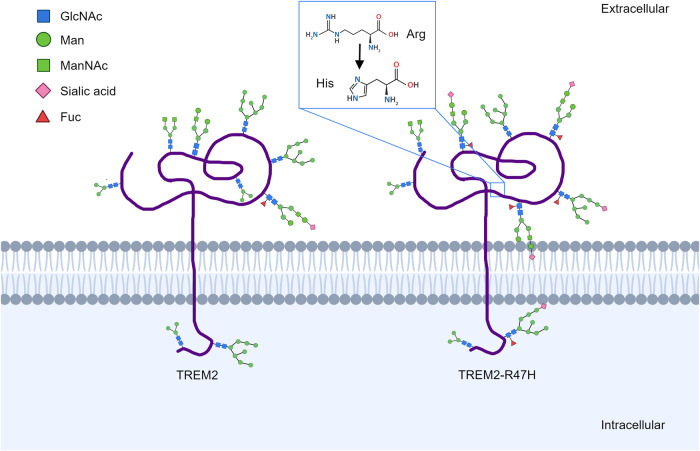
Altered glycosylation patterns of TREM2-R47H in AD. The expression of TREM2 is increased in AD and TREM2-R47H, a variant of TREM2, on which the Arg in the 47th position mutates to a His, is thought to increase the risk of AD in human. AD-associated R47H variant of TREM2 increases its terminal glycosylation with complex oligosaccharides, including the non-high mannose type glycan, and fucosylated-sialylated complex/hybrid type glycans, etc. This mutation may alter the ability of TREM2 to bind to ligands and its receptor function.

An R47H-encoding variant of the triggering receptor expressed on myeloid cells (TREM2-R47H) was recently reported to be involved in the pathogenesis of AD. TREM2 is a type 1 membrane protein consisting of an extracellular immunoglobulin-like domain and a short cytoplasmic tail, which may function in microglial cells and myeloid cells such as osteoclasts (OCs) [85]. TREM2 in microglia is also involved in the clearance of nerve fragments in brain injury, and its expression is increased in AD [86, 87]. There are several mutants of TREM2, of which the R47H variant has an increased expression in AD patients and is thought to increase the risk of AD in human. This may be because this mutation alters the ability of TREM2 to bind to its ligand and the function of the receptor. TREM2-R47H has an altered glycosylation pattern and reduced stability [37]. Nano-LC/MS analysis have shown that AD-associated R47H variant of TREM2 increases its terminal glycosylation with complex oligosaccharides in the Golgi apparatus, including the non-high mannose type glycan, and fucosylated-sialylated complex/hybrid type glycans, etc. (Fig. 5). However, the variants’ stability is reduced at the same time, which may be caused by their decreased solubility, and the decreased solubility may cause the proteasome and lysosomal degradation systems more active to degrade the variants and maintain the normal level of TREM2. Therefore, it is supposed that the glycosylation status of TREM2-R47H may affect its solubility and stability, as well as its translocation to the cell surface [88]. Given that TREM2 protein plays a variety of roles in brain injury repair and other processes, its reduced stability and accelerated degradation may lead to overloading of cellular degradation capacity, causing problems such as ER stress and lysosomal degradation. The restricted transport of TREM2 in cells may affect ligand-binding function of cells, resulting in the accumulation and aggravation of the neurological damages, thereby promoting the pathogenesis of AD.

#### APOE and APOJ

Apolipoprotein E (APOE) and apolipoprotein J (APOJ) have been shown to be associated with the development of Alzheimer’s disease [89, 90]. Interestingly, both APOE and APOJ can act as ligands for TREM2. This binding is reduced in AD [91]. ApoE genotype accounts for the vast majority of AD risk and AD pathology, and APOE fragments are thought to contribute to the formation of amyloid plaques and neurofibrillary tangles in AD [90]. As an O-glycosylated protein [92], glycosylation modifications of APOE are thought to enhance its solubility and affect neuronal health. When soluble glycosylated APOE is reduced in the brain, neuronal dendritic spine density increases [93]. In addition, the C-terminal glycosylation of APOE proteins interacts directly with receptor proteins, affecting their binding flexibility, which may be related to sialylation [94]. Three different APOE isoforms, APOE2, APOE3, APOE4, are mainly present in humans, with different glycosylated forms and different effects on AD risk, symptoms and pathology. In plasma and CSF, the levels of APOE2, APOE3, APOE4 glycosylation showed a decreasing trend in sequence [95]. The detection of APOE genotypes and its glycosylation patterns may help to predict the risk of AD in people and assess the progress of AD. A combined analytical model of plasma glycosylation occupancy, total plasma apolipoprotein concentration, and APOE genotype has been shown to accurately differentiate amyloid status [96]. Recent new studies, on the other hand, have confirmed that the percentage of cerebrospinal fluid APOE glycosylation is positively correlated with Aβ42 levels in CSF. Changes in glycosylation levels may affect the ability of APOE to aid in Aβ clearance and degradation through receptor-mediated transcytosis and endocytosis by affecting their binding to HDL. This provides new ideas on how APOE affects the pathogenesis of AD [97].

The concentration of APOJ in AD brain is about 40% higher than that in normal brain, and it is closely related to the formation of senile plaques [89]. A recent study using liquid chromatography-tandem mass spectrometry (LC-MS/MS) to study the glycosylated form of human plasma APOJ confirmed that the APOJ protein is highly glycosylated intracellularly, with a total of six N-glycosylation sites, each with varying degrees of glycosylation diversity. The three glycosylation sites of α64N, β64N and β147N show obvious differences in glycosylation patterns between the samples with low hippocampal atrophy and high hippocampal atrophy. Among them, the specific glycosylation at the β64N site can be used to distinguish low hippocampal atrophy and high hippocampal atrophy samples. Eight β64N glycoforms are significantly reduced in patients with high atrophy compared with those with low atrophy [98]. This result means that it will be possible to assess the level of hippocampal atrophy in patients by detecting the glycosylation pattern of plasma APOJ, thereby judging the progression of AD. Although larger studies are needed to confirm the feasibility of APOJ as a biomarker for AD, APOJ has been considered to have great potential. In the future, targeted SRM technology (selective response monitoring technology for targeted proteomic quantification) may also expand the application of APOJ as a biomarker for AD, enabling the detection of specific glycosylation levels in individual patients to learn about their own level of hippocampal atrophy, and achieve the effect of precision medicine.

**Fig. 6:**
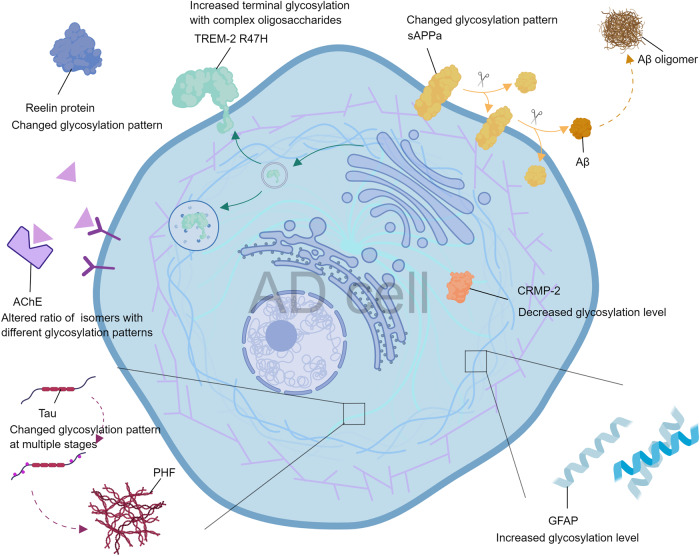
Changes in glycosylation of key AD proteins. In AD patient cells, some proteins have abnormal glycosylation patterns, such as tau protein, APP, GFAP, CRMP-2 and TREM. This figure summarizes most of the proteins mentioned in this review and the altered patterns of their glycosylation. The exact mechanism of the effect of abnormal protein glycosylation on AD is still unclear, but glycosylation is considered to be a novel entry point for understanding AD. The protein glycosylation changes shown in this figure do not necessarily occur simultaneously in the same AD cell; this is only a figurative summary.

**Fig. 7:**
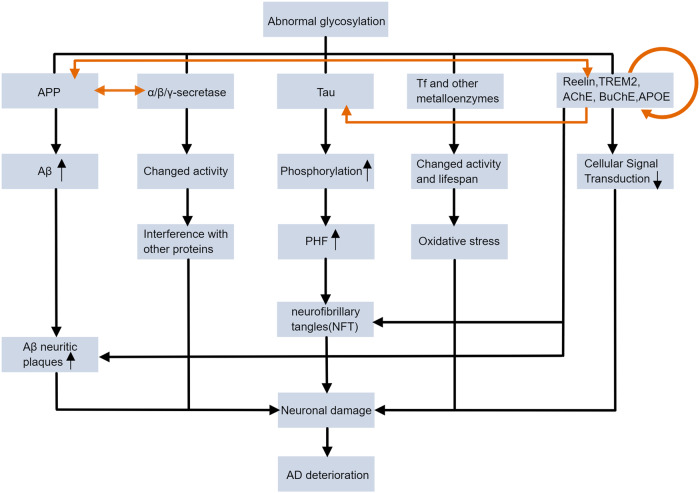
The impact of abnormal glycosylation of proteins on AD pathology. Aberrant glycosylation of APP and its processing enzymes may exacerbate the aggregation of Aβ amyloid plaques. Aberrant glycosylation of tau increases PHF formation by affecting its phosphorylation. Altered glycosylation of Tf and other metalloenzymes affects intracellular oxidative stress by influencing enzyme activity and lifespan. Glycosylation changes of other proteins related to cell signaling, such as TREM2 and Reelin, may affect cell signaling. Together, these changes contribute to neuronal damage in the pathogenesis of AD. The black lines indicate a series of changes following abnormal glycosylation, and the orange lines indicate the interactions between proteins.

We summarized the above AD key proteins for their glycosylation changes in Fig. 6 and Table 2, and we also proposed our currently knowing of the mechanism of aberrant protein glycosylation on the developing of AD in Fig. 7.

**Table 2:** Altered glycosylation patterns in AD.

Protein	Altered glycosylation patterns in AD	Reference
Tau	Increased N-glycosylation at an early stage, decreased O-GlcNAc glycosylation in cytoskeletal fractions and higher levels of O-glycosylation in neurofibrillary tangles	[29, 34, 35]
Reelin protein	Different binding properties to lectins such as *Lens culinaris agglutinin* and *Ricinus communis agglutinin*	[30]
CRMP-2 protein	Decreased level of glycosylation	[16]
GFAP	Increased level of glycosylation	[16]
AChE	Altered ratio of AChE isomers with different glycosylation patterns	[81]
APP	APP that undergoes two different cleavage patterns differs in glycosylation	[43]
Transferrin	Increased TfC1 protein sialylation levels with decreased TfC2 protein sialylation levels	[62–64]
TREM2-R47H	Increased terminal glycosylation with complex oligosaccharides in the Golgi apparatus	[37]
APOJ	Differences in glycosylation patterns of three glycosylation sites of α64N, β64N and β147N	[98]

### Overall changes of N-Glycosylation/O-Glycosylation/O-GlcNAc glycosylation in AD

**Fig. 8:**
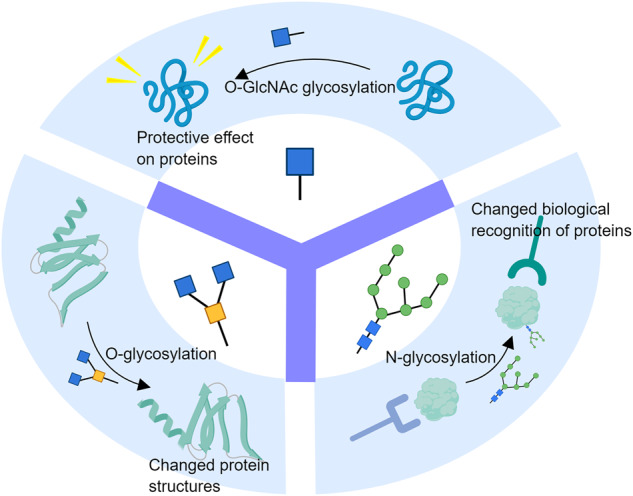
The effects of N-glycosylation/O-glycosylation/O-GlcNAc glycosylation on protein structure and function. N-glycosylation is associated with protein biorecognition and is involved in the processing and translocation of several AD key proteins to the cytosolic membrane. O-glycosylation mainly affects protein structure, and further affects protein properties such as stability and binding capacity. O-GlcNAc glycosylation exhibits a protective effect on proteins. It may help reduce the dynamic changes and enhances the stability of proteins.

Different glycosylation patterns have different effects on proteins and play different roles in the development of AD. Integrative glycoanalysis focused on the overall trend of N-glycosylation, O-glycosylation and O-GlcNAc glycosylation in AD brain. The levels of multiple enzymes involved in N-glycosylation and O-glycosylation are down-regulated in some brain regions that are significantly impaired in AD [99]. The levels of protein O-GlcNAc glycosylation show region-specific changes in AD brain, with overall levels being reduced in AD but increased in the hippocampus [28, 53]. In addition, sialic acid levels in AD are increased in the cerebellum [100]. These findings may suggest an overall correlation between glycosylation patterns and AD development. Exploring the effects of N-glycosylation/O-glycosylation/O-GlcNAc glycosylation on protein structure and function would be of great help in the development of symptomatic AD drugs (Fig. 8). In previous studies, it has been shown that N-glycans are associated with protein biorecognition and are involved in the processing and translocation of APP and TREM2 to the cytosolic membrane [46, 88]. O-glycosylation mainly affects protein structure. The C-terminal O-glycosylation of APOE is associated with the flexibility of its binding receptor [94]. O-glycosylation on APP alters the stability of APP through structural modifying effects and affects the degradation of Aβ proteins [101]. O-GlcNAc, on the other hand, exhibits a significant positive protective effect. In studies concerning synaptic protein I (SynI), it was shown to help reduce dynamic changes in the protein, leading to enhanced stability and protection of neurons [102]. O-GlcNAc glycosylation also regulates the formation of COP II vesicles by modulating important proteins on COP II vesicles. In AD, Aβ proteins affect the directional transport of COP II vesicles by impairing O-GlcNAc glycosylation [103]. O-GlcNAc glycosylation is also able to reduce tau phosphorylation and inhibit PHF production through site competition [53].

In response to the trend of overall changes in the levels of several glycosylations in the AD brain, some diagnostic and therapeutic targets for AD are the overall levels of glycosylation in the AD brain. Experiments targeting the abundance and types of N-glycan in AD serum have revealed its potential to determine the state of AD progression by its altered profile [104]. However, there have been no attempts to use overall altered glycosylation levels as a biomarker for AD pathogenesis. Currently, the main molecular targets for altering overall glycosylation levels are various glycosylation-related enzymes. The molecular structure of O-GlcNAc glycosidase (OGA) has been studied in detail in recent years with the aim of designing the targeted inhibitor [105] for enhancing O-GlcNAc glycosylation levels in AD brain, which may have a protective effect. Backfilling against multiple enzymes involved in N-glycosylation and O-glycosylation that are known to be down-regulated in AD cells is also a potential AD treatment that may ameliorate AD symptoms by boosting the stability of APP, slowing down the processing and transport of aberrant proteins, and in a number of other ways. It has also been proposed that targeting the gene for N-acetylglucosaminyltransferase III (GnT-III) by miR-23b to inhibit its expression can improve AD symptoms [106].

Changes in the intracellular protein glycosylation patterns can also reflect the drug effectiveness. To study whether or not Liuwei Dihuang formula (LW-AFC), which is prepared from the traditional Chinese medicine prescription Liuwei Dihuang decoction, has a curative effect on dementia mice, the glycoproteins in the cerebral cortex and serum of AD model mice in the experimental group and the control group were analyzed and compared with each other. Several N-glycans whose abundance was significantly decreased in AD mice were significantly recovered after LW-AFC treatment, which demonstrated that LW-AFC may improve the symptoms of mice with dementia by regulating N-glycans patterns [107]. Changes in glycosylation patterns before and after dosing may be combined with changes in apparent behavior and pathological characteristics as a measure of the effectiveness of drugs in treating AD, making the evaluation of drugs tend to be multi-level and comprehensive.

While there are many conjectures about the way of glycosylation to play in AD diagnosis and treatment, few of them has been validated and concluded in specific experiments. Glycosylation for AD diagnosis and treatment is a relatively new and promising field, and more researches should be conducted in the future to further develop and explore this field. However, it should be noted that the changes in the glycosylation pattern of proteins in AD are complex, as there may be a negative correlation between protein O-GlcNAcylation and N-/O-glycosylation pathways, indicating that there may be mutual regulation between different glycosylation patterns [28]. When designing the targeted drugs, attention should also be paid to their effects on other intracellular protein glycosylation and glucose homeostasis.

### Conclusion And Perspectives

AD is a progressive neurodegenerative disease, and the basic experimental and clinical researches on AD are being intensively undertaken. As the most common means in post-translational modification of proteins, the role of protein glycosylation in AD has been received increasing attentions. The glycosylation patterns of various key proteins in AD (such as tau, APP, CRMP-2) are found to be altered, suggesting a strong link between protein glycosylation and AD development.

Recently, many experiments on AD glycosylation focus on the mechanisms of protein glycosylation affecting AD pathogenesis. It has been found that changes in glycosylation patterns may affect the processing products of APP protein, alter the degree of abnormal phosphorylation of tau which directly affect the pathological characteristics of AD. Glycosylation also affects cellular oxidative stress by regulating the lifespan and activity of enzymes such as transferrins and Cu, Zn-SOD. The development of glycoproteomics technology will help improve the overall understanding of protein glycosylation in AD, and discover more metabolic pathways involved in abnormally glycosylated proteins in AD.

Proteins with altered glycosylation patterns in AD may serve as biomarkers for AD diagnosis, while how to accurately detect the changes of the glycosylation patterns and levels and how to determine whether the change is a specific change in AD are the key for this application. Targeting specific proteins and altering the levels of their glycosylation are also proposed as potential for developing new drugs for the treatment of AD. However, because the changes in protein glycosylation patterns in AD are complex and interact with each other, special attention should be paid to the possible adverse effects on intracellular glucose homeostasis when designing new drugs.

In the future, changes in glycosylation patterns in AD may be linked to changes in metal ions as a potential new area of research considering the mutual influence and co-action of sugar homeostasis and metal homeostasis in AD. The possible feedback regulation between N-glycosylation and O-glycosylation also deserves further study to gain insight into the formation of intracellular sugar homeostasis. An improved understanding of protein glycosylation patterns in AD will facilitate the development of glycan-targeted AD biomarkers and drug targets to improve the diagnosis and treatment of AD.

## Data Availability

There are no experimental datasets, given that this is a review article that is prepared based on a literature review.
